# Radiolabelled Antisense miRNA Oligonucleotides for Molecular Imaging and Therapeutic Applications

**DOI:** 10.3390/pharmaceutics18101197

**Published:** 2026-09-22

**Authors:** Chiara Bonanno, Giuseppe Romeo

**Affiliations:** 1 Department of Chemical Sciences, University of Catania, 95125 Catania, Italy; 2 Institute of Bioimaging and Complex Biological Systems, National Research Council, 90015 Cefalù, Italy; 3 Department of Drug and Health Sciences, University of Catania, 95125 Catania, Italy; 4 NANOMED—Research Center on Nanomedicine and Pharmaceutical Nanotechnology, University of Catania, 95125 Catania, Italy

**Keywords:** microRNA, antisense oligonucleotides, molecular imaging, radiolabelling

## Abstract

**Background/Objectives:** MicroRNAs (miRNAs) are small non-coding RNAs (20–24 nucleotides) that regulate post-transcriptional gene expression and are involved in key biological processes, including proliferation and apoptosis. Dysregulation of miRNA expression is associated with numerous diseases, particularly cancer, making them attractive biomarkers and therapeutic targets for precision medicine. Antisense miRNA oligonucleotides (AMOs) have emerged as promising agents for selectively inhibiting miRNA activity. This review examines the development of radiolabelled AMOs targeting miRNAs for diagnostic and imaging applications, with particular attention to their radiolabelling, biological performance, delivery strategies, and potential therapeutic extension. **Methods:** The literature was examined with a focus on studies describing the synthesis of radiolabelled AMOs targeting miRNAs. Other oligonucleotide-based molecules, such as antisense oligonucleotides (ASOs) and peptide nucleic acids (PNAs), were not considered. **Results:** Current evidence demonstrates that AMOs can be efficiently radiolabelled with clinically relevant radionuclides, including ^99m^Tc and ^68^Ga, using bifunctional chelating agents (BFCA) such as MAG3, DOTA, and NOTA while preserving their biological properties. These probes enable noninvasive imaging of their in vivo localization in regions of interest, supporting their potential for precision diagnostics. However, available studies remain limited, and significant challenges persist, particularly regarding targeted delivery, pharmacokinetics, and nonspecific uptake in excretory organs. Therapeutic applications remain at an early preclinical stage, with radioiodinated AMO-21 providing preliminary evidence for therapeutic use. **Conclusions:** Radiolabelled AMOs represent a potential approach for miRNA targeted molecular imaging, but their clinical translation remains challenging. Future studies should integrate optimized chemical modifications and delivery systems with rigorous in vivo validation and appropriate controls.

## 1. MicroRNAs

MicroRNAs (also called miRNAs or miRs) are short non-coding sequences of RNA molecules. Typically, the mature miRNA length is between 20 and 25 bases. The first miRNA, Lin-4, was discovered in 1993 by Ambros and Ruvkun in *Caenorhabditis elegans* [1], a small nematode. It was necessary to wait until 2000 for the identification of a second miRNA, let-7, a 21-mer long miRNA discovered by Reinhart et al. [2] in the same organism. In the following years, thousands of miRNAs have been discovered in both animal and plant species. Nowadays, more than 2000 different miRNAs involved in various biological processes have been identified in humans [3]. Their crucial role has been recognized at the highest level: in 2024, Victor Ambros and Gary Ruvkun were awarded the Nobel Prize in Physiology or Medicine for the discovery of microRNAs and their role in post-transcriptional gene regulation.

MiRNAs carry out their biological functions only after a process of biogenesis and maturation that can be divided into two main steps: the first step takes place in the nucleus, and the second takes place in the cytoplasm (Figure 1). In the first step, the miRNA gene is transcribed by RNA polymerase II, forming a hairpin intermediate known as pri-miRNA (primary miRNA). This intermediate is recognized by the Microprocessor complex, composed of RNase III endonuclease Drosha and its RNA binding cofactor, DGCR8 (DiGeorge syndrome Critical Region gene 8 protein). DGCR8 recognizes the characteristic stem–loop structure of the pri-miRNA and positions Drosha to cleave the RNA approximately 11 base pairs from the stem–single-stranded RNA junction, generating the pre-miRNA (precursor miRNA), which is about 70 nucleotides long. Then, the pre-miRNA is transported into the cytoplasm thanks to nucleocytoplasmic transporter Exportin 5, which uses Ran-GTP as an energy source. Ran is a small GTPase that acts as a molecular switch, regulating the transport of proteins and RNAs between the nucleus and cytoplasm thanks to the GTP/GDP cycle.

The maturation of the pre-miRNA continues in the cytoplasm. Here, it is processed by RNase III endonuclease Dicer, which associates with RNA-binding protein TRBP (Transactivation Response Element RNA-binding Protein) [4]. Dicer cleaves the pre-miRNA near the terminal loop to generate an approximately 22-nucleotide miRNA duplex composed of the guide strand (the one that will become the mature miRNA) and the passenger strand (miRNA*). Following strand selection, the guide strand is preferentially retained in Argonaute (Ago), whereas the passenger strand is generally released and degraded. However, in some cases, both strands can be retained and function as mature miRNAs [5,6].

The mature miRNA plays a fundamental role in the regulation of gene expression at the post-transcriptional level. In fact, mature miRNA is loaded onto the RISC (RNA-induced silencing complex) protein complex in which the interaction with the target messenger RNA (mRNA) takes place. MiRNA binding generally involves partial complementarity, particularly within the seed region, and can lead to translational repression and/or mRNA destabilization and degradation. For this reason, some miRNAs regulate a large number of target mRNAs, and some of these miRNAs show strictly tissue-specific expression [7].

The function of many miRNAs has not yet been defined, and it is not clear which biological pathways they regulate. Nevertheless, the participation of some miRNAs in various biological processes, such as cell proliferation and differentiation [8,9], apoptosis [10] and angiogenesis [11], has been proven. Furthermore, tissue-specific miRNAs have been identified. Examples are miR-124, specific to the brain and involved in the regulation of dendritic spine development [12], and miR-133a-1 and miR-133a-2, which are specific to the myocardium and essential for the regulation of muscle development, whose absence causes lethal ventricular septal defects in half of embryos [13]. MiRNAs are also used for signal transmission. They are transported from one cell to another via exosomes, small vesicles of endocytic origin that are released into the extracellular environment [14]. It is evident from the above that miRNAs are involved in the regulation of different cellular functions and alteration of their physiological expression can contribute to the development of various pathological conditions.

## 2. MiRNAs in Human Diseases

The involvement of miRNAs in human diseases has attracted increasing attention in recent years, leading to extensive investigation of their potential role in disease diagnosis, prognosis and treatment. In particular, disease-associated changes in miRNA expression profiles have been investigated as potential biomarkers, as well as possible targets for therapeutic intervention [15].

In the cardiovascular field, extensive studies have highlighted that alterations in the levels of specific miRNAs are closely associated with conditions such as cardiac hypertrophy, myocardial infarction, and heart failure. MiRNAs such as miR-208b, miR-499, and miR-1 have been identified as potential non-invasive biomarkers easily detectable in the blood. They enable the early diagnosis and monitoring of heart disease progression, thereby improving clinical patient management [16,17]. Similarly, for kidney disease, numerous studies have identified miRNAs as promising biomarkers for prevalent conditions such as diabetic nephropathy, acute and chronic renal failure, and polycystic kidney disease. Their detectability in urine and blood allows for the early identification of pathological changes in the kidneys and enables the monitoring of disease progression with greater precision than traditional methods [18,19]. Beyond cardiovascular and renal disorders, miRNAs are also implicated in vascular alterations and diabetic complications, where they modulate inflammatory processes and endothelial function. Their ability to regulate inflammatory responses and contribute to vascular dysfunction makes them targets for specific therapeutic interventions and tools for early diagnosis, potentially improving the prognosis of chronic vascular diseases [20,21,22].

Among the different pathological conditions in which miRNA dysregulation has been investigated, cancer represents one of the most extensively studied fields. It is already known that tumour cells produce different types and quantities of miRNAs compared to the corresponding healthy cells from which they originate [23,24].

In general, it is not possible to give a generic definition of the role of miRNA role tumours. Indeed, it is complex to classify their function in a unique way because each miRNA in each type of tumour plays a different role. An example concerns chronic lymphocytic leukaemia (CLL), characterized by a prevalence of non-dividing malignant B cells that overexpress the antiapoptotic B-cell lymphoma 2 (Bcl-2) protein. In this type of tumour, miR-15a and miR-16-1 are underexpressed or completely absent in tumour cells. These miRNAs target the antiapoptotic *Bcl-2* gene and regulate its expression in healthy cells. Therefore, the lack of these miRNAs leads to an inhibition of apoptosis and, consequently, to an increase in the malignancy of these tumours. Thus, the role of miR-15a and miR-16-1 as tumour suppressors is evident [25]. Conversely, miR-186 acts like a tumour promoter in cutaneous squamous cell carcinoma (cSCC). The target of this miRNA is Apoptotic Protease Activating Factor 1 (APAF1). In cSCCs, miR-186 is overexpressed, leading to the downregulation of *APAF1* gene expression. This causes cell proliferation, invasion and migration, as well as inhibition of cellular apoptosis. These types of miRNAs, which promote tumour development, are called oncomiRNAs [26].

It is clear, therefore, how the expression profile of miRNAs varies according to the type of tumour and how it is different from the physiological expression of miRNAs in healthy cells. From this perspective, miRNAs represent a potential new diagnostic and prognostic tool in the field of oncology.

This application becomes even more interesting considering that the alterations of miRNA expression are also reflected in biological fluids, such as plasma, serum and urine.

A study by Bocchini et al. [27] demonstrates that pancreatic neuroendocrine tumour (PanNET) patients with ^18^F-FDG-PET/CT positivity show a significant alteration in the expression of three miRNAs in plasma: hsa-miR-5096, hsa-miR-4311 and hsa-let-7i-3p. These were studied as predictors in the PanNETs individually, in pairs and as a single–triple combination. The study seems to be only a preliminary study based on a small number of patients but appears to be very promising.

Even in urine, it is possible to evaluate alterations in the production of miRNAs by tumour cells. A study conducted by Xuan-Mei Piao et al. [28] explored the possibility of distinguishing non-malignant haematuria from that associated with bladder cancer by analysing free miRNAs present in urine. A total of 543 samples were examined, highlighting that in patients with bladder cancer, the ratio between miR-6124 and miR-4511 is significantly higher than that in patients with non-malignant haematuria, with a diagnostic precision of 90%. This approach can reduce the number of unnecessary cystoscopies in patients with haematuria, thereby improving the diagnostic process.

MiRNAs also have great potential as therapeutic agents in oncology. It is sufficient to consider that they can regulate various miRNA-dependent signalling pathways, amplifying or inhibiting them where necessary. There are two ways to exploit miRNAs for therapeutic purposes. The use of one over the other depends on how miRNA expression is modulated from the onset of the disease.

If a miRNA is underexpressed in a tumour, contributing to its development or increased aggressiveness, mimics can be used. Mimics are synthetic oligonucleotides that have the same function as endogenous miRNAs, sharing the same molecular target. They are called “mimics” because they mimic the action of natural miRNAs, reproducing their behaviours [29].

Conversely, if a miRNA (oncomiRNA) is overexpressed and promotes tumour growth, an Antisense MiRNA Oligonucleotide (AMO) can be used to block the oncomiRNA. In this way, the signalling pathway regulated by that miRNA is silenced, interrupting the miRNA’s contribution to the development of the disease [30,31].

## 3. Antisense miRNA Oligonucleotides

Antisense miRNA oligonucleotides are synthetic DNA or RNA sequences approximately 15–24 nucleotides long. As their name suggests, they are sequences complementary to the mature miRNA target, designed to specifically bind it, blocking its function.

There are three fundamental requirements that AMOs must satisfy to be used as diagnostic and therapeutic tools for miRNA inhibition. The first concerns the AMO’s ability to reach the target in the intact form. Specifically, the AMO must survive enzymatic degradation (for example, by nucleases) and be able to cross the cell membrane to interact with the miRNA inside the cell. The second point concerns the binding affinity between AMO and miRNA, which is directly related to the stability of the AMO–miRNA duplex. This stability is measured by the melting temperature (Tm) of the duplex: the higher Tm is, the more energy that will be needed to break the bonds between the two nucleotide sequences. Finally, it is essential that the AMO does not cause toxicity or serious side effects in the patient [32].

An unmodified antisense miRNA oligonucleotide does not usually meet all the requirements described above. In fact, it would be easily degraded by nucleases, and this drastically reduces the number of molecules available to bind to the target. Furthermore, molecules that reach the target cell intact have difficulty penetrating the cell membrane because they are relatively big, negatively charged, and highly hydrophilic. For this reason, modified AMOs, designed to overcome these limitations, are generally used.

The modifications most commonly used involve the phosphodiester bond (PO) and the sugar moiety of the oligonucleotide.

In particular, the PO (Figure 2, structure **1**) is often alternated or completely replaced by phosphorothioate bond (PS) (Figure 2, structure **2**). Replacing a non-bridged oxygen atom with a sulphur atom not only disfavours the attack of nucleases, decreasing the hydrolysis of the oligonucleotide, but also promotes the interaction of the oligonucleotide with both plasma and cellular proteins. All these factors improve pharmacokinetics by increasing the molecule’s permanence in the blood and promoting cellular uptake. However, this increased persistence in the body can also be a drawback, as it may elevate the risk of toxicity [33,34].

The modifications to the sugar moiety mainly concern the hydroxyl group in the 2′ position. During synthesis, this group must necessarily be protected to avoid secondary reactions. Generally, instead of using temporary protective groups to be removed at the end of the synthesis, chemical modifications are introduced, which are retained in the final oligonucleotide and can confer additional functional advantages. The most commonly used groups are 2′-*O*-methyl (2′OMe) and 2′-*O*-methoxy-ethyl (2′MOE) (Figure 2, structures **3** and **4**), which are bulkier than OH, creating a steric hindrance, which hinders the action of nucleases, but without being so bulky as to interfere with the interaction between AMO and miRNA. Rather, these groups have been shown to enhance complementary oligonucleotide–miRNA binding affinity [35].

Another frequently used modification is represented by Locked Nucleic Acids (LNAs) (Figure 2, structure **5**), in which the oxygen in the 2′ position is linked to the carbon in the 4′ position via a methylene bridge. These modified nucleotides offer remarkable hybridization capacity and excellent resistance to nuclease attack [36].

The modifications discussed above are just some of the existing ones and can be used in any desired combination to modify the characteristics of the oligonucleotide being synthesized.

An innovative strategy still under investigation is the radiolabelling of AMOs. In this context, the aim of this paper is to provide an overview of the current literature on radiolabelled AMOs targeting miRNAs for molecular imaging and therapeutic purposes. Other classes of antisense oligonucleotides, including conventional Antisense Oligonucleotides (ASOs) and Peptide Nucleic Acids (PNAs), as well as oligonucleotide-based probes targeting RNA species other than miRNAs, are beyond the scope of this review. Within this defined scope, we focus on the advantages of this approach, highlighting its limitations, and analysing the potential future developments in the field.

## 4. Radiolabelled AMOs

Radiolabelled AMOs are oligonucleotides antisense to a target miRNA that are labelled with a radioisotope. The antisense oligonucleotide is the carrier that delivers the radiotracer to the target miRNA. The radioisotope, thanks to the radiation it emits, gives to the molecule its diagnostic or therapeutic value [37].

Therefore, we can easily classify radioisotopes used in nuclear medicine based on the type of radiation emitted during decay: γ or β^+^ emitters are used for diagnostic and imaging purposes, while β^−^ and α emitters are used as therapeutic tools. We will see how these radiations work.

γ-rays consist of high-energy photons. Having neither mass nor charge, they have a high tissue penetration capacity. This characteristic allows them to pass through the human body so that they can be identified by a detection system. The medical equipment used with γ-ray emitted radioisotope is the SPECT (Single Photon Emission Computed Tomography) instrument. It typically consists of scintillators coupled with photomultiplier tubes. The detectors convert the gamma photons into visible/UV light photons, then into electrical signals, allowing the system to reconstruct detailed images of the tracer distribution within the body. When this technique is combined with computed tomography, 3D morphological imaging can be acquired. Indeed, it is widely used for tumour staging, preoperative imaging, and re-staging of the tumour after surgery and/or medical treatment. An example of the most widely used γ-emitter radionuclide in nuclear medicine is technetium-99m (^99m^Tc), which decays by emitting detectable photons with an energy of 140 keV, and it has a half-life of approximately 6 h [38].

For the same purpose, β^+^-emitter radioisotopes are used. β^+^ particles, also called positrons, have the same mass and the same but opposite charge as electrons. Therefore, they are positive particles. When positrons, emitted following the radiative decay of an unstable atom, encounter an electron, an annihilation reaction occurs. This reaction leads to the formation of two γ-rays in opposite directions. These two gamma rays can be detected by PET (Positron Emission Tomography) instruments. Compared to SPECT, PET does not detect the emitted photons directly from the radionuclide but the coincident pairs of γ-rays resulting from positron–electron annihilation. This coincidence detection allows for more accurate event localization, providing higher sensitivity and spatial resolution than SPECT. Among the β^+^-emitters, gallium-68 (^68^Ga) and fluorine-18 (^18^F) are definitely worth mentioning. Both are widely used for the production of radiopharmaceuticals with diagnostic purposes [39].

β^−^ particles are high-energy electrons. They have a small mass and negative charge. β^−^ particles have a lower penetrating capacity than gamma rays and travel over relatively short distances in biological tissues (mm). In fact, due to their mass, they interact with the molecules they encounter along their path, releasing energy. This leads to direct and indirect damage to the cells. Direct damage refers to the damage these particles can cause directly to DNA (typically, single-strand breaks that are reversible), while indirect damage concerns the ionizing capacity of these particles. β^−^ particles are capable of ionizing water molecules in the cytoplasm of target cells, leading to the formation of free radicals. These highly reactive radicals can, in turn, damage various cellular structures, such as proteins and lipid membranes. For this reason, radioisotopes that decay by emitting β^−^ particles are used for therapeutic purposes. Lutetium-177 (^177^Lu) decays mainly by emitting β^−^ radiation, and it is one of the most widely used radionuclides in oncological therapies, especially for neuroendocrine and prostate tumours [40,41]. Another relevant example is iodine-131 (^131^I), which emits both β^−^ particles, contributing to its therapeutic effect, and γ-rays, allowing for its use for imaging and dosimetric purposes [42].

α particles are formed by two protons and two neutrons. They are the heaviest particles among those mentioned above, which gives them a low penetration capability (μm). When delivered inside cells, they can cause highly localized and complex DNA damage, including double-strand breaks that are more difficult to repair. In addition, like β^−^ particles, α particles can induce indirect damage to tumour cells. Due to their short range, the use of α-particles can limit side effects in surrounding healthy tissues while increasing the efficacy of the radiopharmaceutical [43,44]. Examples of α-emitter radionuclides are radium-223 (^223^Ra) and actinium-225 (^225^Ac) [45,46].

Depending on the type of radioisotope and its chemical characteristics, the radioisotope can be bound to the oligonucleotide in different ways. Based on this, we can group the major radioisotopes used in nuclear medicine into two groups: non-metallic and metallic radioisotopes.

When non-metallic radionuclides such as ^18^F are used, the radioisotope is generally bound to the carrier molecule via a direct covalent bond. An example of this approach is ^18^F-FDG (fluorodeoxyglucose), the most widely used radiopharmaceutical in PET diagnostics. This molecule is a glucose analogue in which the hydroxyl group at the 2 position of glucose is replaced by a radioactive ^18^F atom [47].

In the case of metal radioisotopes such as ^99m^Tc and ^68^Ga, a bifunctional chelating agent (BFCA) is required. The BFCA acts as a linker between the carrier molecule—in this case, the AMO—and the radioisotope. Bifunctional chelating agents are so called because they perform a dual function: on one hand, they covalently bind the carrier molecule; on the other hand, they are able to chelate the metal radionuclide into an inert complex. The stability of this chelate is the main characteristic of these systems. In fact, metal dissociation can lead to a loss of image quality in the case of diagnostic use and an increase in side effects in the case of therapeutic use [48].

Metallic radionuclides exhibit very different chemical characteristics, such as coordination number, ionic radius, and charge, which directly influence the stability of the complexes they form. For this reason, various types of bifunctional chelating agents have been developed over time [49]. The most commonly used are macrocyclic chelators, such as DOTA (1,4,7,10-tetraazacyclododecane-1,4,7,10-tetraacetic acid), NOTA (1,4,7-triazacyclononane-1,4,7-triacetic acid) and NODAGA (1,4,7-triazacyclononane-1-glutaric acid-4,7-acetic acid), which form highly stable complexes with radionuclides with high coordination numbers (e.g., ^177^Lu, ^68^Ga). Other examples are acyclic chelators such as DTPA (diethylenetriaminepentaacetate) or HBED (*N*,*N*’-bis(2-hydroxybenzyl)ethylenediamine-*N*,*N*’-diacetic acid), which are more flexible and faster at binding the metal radioisotope. HBED, in particular, is widely used for the complexation of ^68^Ga in the preparation of ^68^Ga-based radiopharmaceuticals, while DTPA has been extensively employed for the coordination of ^99m^Tc [50].

## 5. Radiolabelled AMOs for Diagnostic Purposes

Currently, the radioisotopes used for labelling of antisense miRNA oligonucleotides are mainly ^68^Ga and ^99^^m^Tc, which are two of the most widely used radioisotopes in nuclear medicine for diagnostic applications. Since these radioisotopes have different characteristics and chemical properties, different oligonucleotide conjugation systems, radiolabelling strategies and reactions have been developed depending on the isotope used (Table 1).

### 5.1. ^99m^Tc Radiolabelled AMOs

^99m^Tc is widely used in FDA-approved radiopharmaceuticals for diagnostic imaging of various human organs, such as the brain, lungs, and heart. ^99m^Tc is a metastable nuclear isomer of technetium-99. It has a half-life of approximately 6 h and decays by emitting readily detectable γ rays with a photon energy of 140 keV. ^99m^Tc is produced using a ^99^Mo/^99m^Tc generator, from which it elutes as sodium pertechnetate (Na^99m^TcO_4_), allowing for on-site production immediately before use. Due to its characteristics and the widespread use of this radionuclide in diagnostic imaging with SPECT [51,52,53], ^99m^Tc was one of the first radionuclides to be used in AMO radiolabelling studies.

Over the last 12 years, the research group of L. Kang, from the Department of Nuclear Medicine, Peking University, has focused on studying the synthesis of AMOs radiolabelled with ^99m^Tc.

The first AMO they synthesized was an antisense oligonucleotide of miR-21 [54]. MiR-21 is a highly conserved, abundantly expressed miRNA that regulates gene expression and plays major roles in cancer, cardiovascular disease and inflammation. MiR-21 is consistently overexpressed in many cancers and acts as an oncomiRNA by downregulating tumour suppressor genes, promoting cell proliferation, survival, invasion, metastasis, and therapy resistance [55,56].

**Table 1:** Radiolabelled AMOs targeting miRNAs for molecular imaging.

Radio- Nuclide	Target miRNA	Oligonucleotide Chemistry	Chelator	Radiolabelling Conditions	Radiochemical Yield	Refs.
**^99m^** **Tc**	miR-21	2′OMe + PS modifications	MAG3 at 5′-end	RT, 90 min, SnCl_2_ reduction	97%	[54]
**^99m^** **Tc**	miR-21	2′OMe + PS modifications	MAG3 at 5′-end	T = 100 °C, 30 min, SnCl_2_ reduction	97%	[57]
**^99m^** **Tc**	miR-155	2′OMe + PS modifications	MAG3 at 5′-end	T = 100 °C, 30 min, SnCl_2_ reduction	97–99%	[58]
**^99m^** **Tc**	miR-21	2′OMe + PS modifications	GAGK peptide at 5′-end	T = 100 °C, 30 min, SnCl_2_ reduction	90%	[59]
**^68^** **Ga**	miR-15b	All 2′OMe nucleotides	NOTA/DOTA derivatives at 3′-end	T = 100 °C, 10 min	Not indicated	[60]
**^68^** **Ga**	miR-15b	All 2′OMe nucleotides	NOTA/DOTA derivatives at 3′-end	T = 95 °C, 10–15 min	96%	[61]

Kang et al. [54] proposed interesting modifications to the structure of the AMO of miR-21 (AMO-21), 22-mer long, to improve nuclease resistance and binding affinity. Considering the sequence of mature miR-21 (5′-UAGCUUAUCAGACUGAUGUUGA-3′), AMO-21 was designed as its complementary antisense sequence. Specifically, it was synthesized with three 2′OMe bases and six bases with PS linkages at each end.

To radiolabel this oligonucleotide with ^99m^Tc, a bifunctional chelating agent, MAG3 (*S*-acetylmercaptoacetyltriglycine), was used. To do this, a primary amine was first added to the AMO-21 5′ end via a six-carbon methylene linker with a terminal amino functional group. The addition of this group at the end of the oligonucleotide allowed the *S*-Acetyl-NHS-MAG3 (*N*-hydroxysuccinimidyl derivative of *S*-acetylmercaptoacetyltriglycine) (Figure 3 compound **6**) to react easily with the oligonucleotide, which incorporated the chelating moiety to give MAG3-AMO-21.

During the conjugation reaction, the sulphur atom remains protected by the acetyl group. Once the conjugation is complete, this protecting group is removed by adding SnCl_2_⋅2H_2_O and heating for 15–20 min, converting the *S*-Acetyl-MAG3-AMO conjugate into the MAG3-AMO conjugate. The molecule obtained as such was ready to be radiolabelled. The structure of MAG3—in particular, the nitrogens of the three amide groups and the sulphur of the thiol group—provides four coordination sites to chelate ^99m^Tc.

To synthesize ^99m^Tc-MAG3-AMO-21 (Figure 4, compound **7**), lyophilized MAG3-AMO-21 was solubilized in sodium tartrate buffer. Then, a solution of SnCl_2_·2H_2_O in ascorbic acid was added to MAG3-AMO-21 solution immediately after ^99m^Tc-pertechnetate. The radiolabelling reaction was carried out at room temperature for 90 min.

SnCl_2_ plays an important role in this reaction as a reducing agent towards ^99m^Tc-pertechnetate. Then, tartrate is used as a transchelator, keeping Tc available to be complexed by MAG3. SnCl_2_, however, easily oxidizes in contact with air, losing its reducing properties, so ascorbic acid is used as an antioxidant.

To optimize the radiolabelling conditions, the following parameters were evaluated: the amount of SnCl_2_, the amount of ^99m^Tc-pertecnetate and the reaction time. At the end, it was concluded that the best radiolabelling yield, equal to 97% (*n* = 5), was obtained by using 2 μg/μL SnCl_2_∙2H_2_O, 5 μL ^99m^Tc-pertecnetate and 90 min as reaction time.

A prolonged radiolabelling reaction could pose a challenge to the practical application of the radiopharmaceutical. In fact, the radiopharmaceutical, when administered and to be fit for purpose, must have a specific activity that depends on many factors, including the employed radionuclide (type of emission and half-life); the pharmaceutical carrier itself (e.g., small molecule, protein, oligonucleotide, or antibody), which determines the biodistribution kinetics; the part of the body to be examined; the sensitivity of the imaging system; and the duration of the examination. In this context, a radiolabel reaction 90 min long could be problematic, considering the half-life of the radionuclide, the overall time required for synthesis, quality-control analysis and transportation of the radiopharmaceutical from the production place to the administration place.

For this reason, subsequent studies by the Kang research group focused on optimizing the synthesis to reduce reaction times while still maintaining a high yield [57]. Synthetic conditions similar to those of the previous work were used, but the radiolabelling reaction took place under a heating condition (about 100 °C) and for a shorter time of 30 min. Increasing the temperature in the radiolabelling reaction allows for a considerable reduction in reaction times. This idea was probably initially discarded due to concerns about stability of oligonucleotides with higher temperature and their potential degradation. However, PS linkages and 2′OMe sugar modifications allow ^99m^Tc-MAG3-AMO-21 to display enhanced stability.

In fact, in [57], Kang et al. demonstrated that ^99m^Tc-MAG3-AMO-21 has not undergone any degradation during radiolabelling reaction. In agarose gel electrophoresis, the bands corresponding to ^99m^Tc-MAG3-AMO-21 before and after radiolabelling are found at the same position. Furthermore, autoradiography of the gel shows a radioactive band at the same position as the synthesized oligonucleotide, confirming successful radiolabelling. With the shorter radiolabelling protocol, they obtained a radiochemical yield of 97% and a radiochemical purity of 99%. Thus, shorter synthesis times led to a probe with higher radioactivity and, therefore, a higher signal useful for the successive in vivo studies.

The stability of synthesized AMO was also studied in human serum. No significant degradation was detected during the 12 h incubation.

Since ^99m^Tc-MAG3-AMO-21 showed promising behaviour in in vitro tests, Kang et al. studied its biodistribution in vivo. In particular, the biodistribution of ^99m^Tc-MAG3-AMO-21 and ^99m^Tc-MAG3-control (an oligonucleotide with a scramble sequence with no interaction with any human gene) was studied in HeLa tumour-bearing mice.

Significant differences were found between the behaviours of the two probes, particularly in the Tumour/Non-Tumour (T/NT) ratio. This parameter represents a quantitative index that compares tracer uptake in tumour tissue to a non-tumour reference area in SPECT images. The T/NT ratio allows for the evaluation of lesion-background contrast, and high T/NT ratios improve diagnostic interpretation accuracy. Comparing the T/NT ratio of the two probes, ^99m^Tc-MAG3-AMO-21 showed a progressive increase over the 10 h monitoring period, with the exception of the kidneys. The ratio reached a maximum at 8 h (from 1.83 at 0.5 h to 5.23 at 8 h) before declining to 4.8 at 10 h. In contrast, the T/NT ratio of the ^99m^Tc-MAG3-control probe remained nearly constant throughout the entire time course, ranging from 1.11 to 1.06 at 10 h. The T/NT ratio of the control probe was consistently lower than that of ^99m^Tc-MAG3-AMO-21. These results suggest a preferential accumulation of ^99m^Tc-MAG3-AMO-21 in tumour tissue compared with the control probe. This supports the potential of ^99m^Tc-MAG3-AMO-21 as a specific probe for miRNA-21 imaging.

The same radiolabelling method was also applied on another miRNA: miR-155 [58]. It is known that miR-155 is involved in the oncogenesis processes of breast, lung, liver and lymphatic system tumours. In all these types of tumours, overexpression of this miRNA has been highlighted [62].

The synthesis of ^99m^Tc-MAG3-AMO-155, 23-mer long, employed the same conditions as the synthesis of ^99m^Tc-MAG3-AMO-21. Moreover, the two oligonucleotides were synthesized with the same modifications (2′OMe bases and PS linkages).

The oligonucleotide radiolabelling process afforded a high yield (consistently above 97%, *n* = 5), with a radiochemical purity of 99% (*n* = 5), which is comparable to that obtained for ^99m^Tc-MAG3-AMO-21 (ranging from 99.1% to 95.8% over 12 h). These findings confirm the efficacy of the previously optimized synthesis method, even on oligonucleotides different from AMO-21.

Initially, to evaluate the impact of the structural modifications on the stability of ^99m^Tc-MAG3-AMO-155, a second antisense oligonucleotide sharing the same sequence but lacking the 2′OMe and PS modifications was synthesized—namely, ^99m^Tc-MAG3-AMO-155*. The synthesis and radiolabelling protocol were the same as those used for ^99m^Tc-MAG3-AMO-155. The stability of both compounds was monitored in human serum over a 12 h period, sampling at 2 h intervals. Gel electrophoresis analysis confirmed the excellent stability of the modified oligonucleotide, which showed no signs of degradation. In contrast, the unmodified oligonucleotide, ^99m^Tc-MAG3-AMO-155*, showed significant diffusion in the gel, indicative of a degradation and thereby confirming that the structural modifications confer superior resistance to nuclease-mediated enzymatic hydrolysis.

Having confirmed the compound’s stability, cellular uptake tests were carried out in MCF-7 cells with ^99m^Tc-MAG3-AMO-155 and ^99m^Tc-pertechnetate. The ^99m^Tc-MAG3-AMO-155 cellular uptake continued to increase (from 2% to 16.2%, *n* = 4) during the 10 h of incubation, while the uptake relative to ^99m^Tc-pertechnetate remained relatively low and stable—3% (*n* = 4). This indicates that a fraction of the ^99m^Tc-MAG3-AMO-155 is able to penetrate the cells, even without the need for an appropriate delivery system.

^99m^Tc-MAG3-AMO-155 was also tested in vivo to check its biodistribution. ^99m^Tc-MAG3-AMO-155 and ^99m^Tc-MAG3-control (a scramble sequence of AMO-155) were injected into MCF-7 tumour-bearing mice. The T/NT ratio of the ^99m^Tc-MAG3-AMO-155 was significantly higher than that of ^99m^Tc-MAG3-control (*p* < 0.01) (except for kidneys and the liver). These data indicate an enhanced tumour uptake of the AMO sequence compared to the scramble control. Further studies are required to fully elucidate the contribution of specific miRNA-155 binding versus non-specific tissue retention.

Despite the good results obtained in biodistribution experiments on tumour-bearing mice, there is still wide room for improvement. In fact, from the biodistribution tests of ^99m^Tc-MAG3-AMO-155 and ^99m^Tc-MAG3-AMO-21, it is possible to see an intense accumulation of radioactive probes in the abdominal region—in particular, in the liver and kidneys, which indicates clearance by the urinary and hepatobiliary systems. This marked nonspecific uptake by the organs responsible for metabolism and elimination results in a high background signal that can mask or make the signal associated with tumour tissue difficult to distinguish, especially in the case of neoplasms located in the same anatomical region. Consequently, the low T/NT ratio compromises imaging quality and diagnostic accuracy. It is worth noting that a direct quantitative comparison between the two probes is limited by their different biological targets (miR-21 vs. miR-155) and the distinct tumour models in which they were evaluated (HeLa vs. MCF-7 xenografts). Nevertheless, evaluating their individual performance profiles provides valuable insights into their respective in vivo behaviours.

As detailed in Table 2, both tracers successfully accumulated in their target tissues, though with different retention kinetics. ^99m^Tc-MAG3-AMO-21 achieved a tumour uptake of 2.11 ± 0.42% ID/g (Injected Dose per gram of tissue) at 0.5 h in HeLa tumours, which declined to 0.91 ± 0.07% ID/g at 8 h. Conversely, in the MCF-7 model, ^99m^Tc-MAG3-AMO-155 showed a higher initial accumulation (5.17 ± 1.07% ID/g at 1 h) and a remarkably stable long-term retention, remaining at 3.72 ± 0.43% ID/g after 8 h. This robust retention within the MCF-7 microenvironment allowed the ^99m^Tc-MAG3-AMO-155 probe to reach a tumour-to-blood (T/B) ratio of 4.74 ± 0.59 at the final timepoint, whereas ^99m^Tc-MAG3-AMO-21 achieved a T/B ratio of 2.52 ± 0.56.

However, despite these promising individual target-to-blood dynamics, the overall T/NT contrast remains compromised by the substantial abdominal background observed for both systems. Therefore, it would be important to develop strategies able to improve the specific accumulation of the probe in tumour cells to increase image contrast, enable clear tumour identification, and reduce unwanted uptake in healthy tissue.

It is noteworthy that the radiolabelling strategy described above was recently applied to tofersen (Qalsody), an ASO approved for the treatment of amyotrophic lateral sclerosis. Tofersen was, in fact, conjugated to the MAG3 chelator and radiolabelled with ^99m^Tc. In human SPECT studies, the ^99m]^Tc-MAG3-tofersen tracer mirrored the distribution of the unlabelled drug [63].

To address this problem, a new study was conducted by the Chen group [59], with the aim of reducing the probe’s permanence in the abdominal region and obtaining better SPECT images. They took inspiration from a previous study of Vanbilloen et al. [64] concerning some bifunctional peptidic chelating agents used to trap ^99m^Tc. This study had reported how tetrapeptide chelators—in particular, GAGG (Gly-Ala-Gly-Gly)—are able to efficiently chelate ^99m^Tc. Moreover, the ^99m^Tc-GAGG complex in mice and baboons had shown plasma clearance similar to that of ^99m^Tc-MAG3 but with lower renal retention. Furthermore, ^99m^Tc-GAGG also had slightly lower uptake in the liver and intestine than its analogous chelate with MAG3.

Therefore, Chen et al. used a tetrapeptide as a bifunctional chelator to study how this influences the hybridization of the AMO with the target miRNA and the AMO biodistribution in vivo.

Having already accumulated a large amount of data in previous studies, AMO-21 was used as the object of this study, and GAGK was chosen as a bifunctional chelating agent. GAGK is a tetrapeptide made up of Glycine, Alanine, Glycine and Lysine.

^99m^Tc was the radionuclide, and the radiolabelling reaction was performed under experimental conditions similar to those used for the preparation of ^99m^Tc-MAG3-AMO-21 [49]. However, using a different chelator, a fine tuning of the radiolabelling parameters was necessary. The SnCl_2_·2H_2_O concentration, reaction temperature and time were re-evaluated.

First, the temperature and the reaction time were studied. It was necessary to evaluate them simultaneously because they are closely related to each other. Therefore, the best radiolabelling conditions to obtain ^99m^Tc-GAGK-AMO-21 (Figure 5, compound **8**) were 100 °C for 30 min. These are also the same conditions that were used in previous studies for radiolabelling of ^99m^Tc-MAG3-AMO-21 and ^99m^Tc-MAG3-AMO-155.

Then, the quantity of SnCl_2_·2H_2_O used to optimize the radiolabelling reaction was studied. Its concentration was varied from 1 to 4 mg/mL (1-2-3-4 mg/mL). From the experimental results, it was evident that the lowest concentration of SnCl_2_·2H_2_O corresponds to the greatest radiolabelling efficiency. Therefore, 1 mg/mL was the chosen concentration.

Once all the parameters of the radiolabelling reaction had been defined, the probe was synthesized, and its stability in serum was monitored for 12 h, with sampling every 2 h. In this case, as was done for ^99m^Tc-MAG3-AMO-21 and ^99m^Tc-MAG3-AMO-155, a gel electrophoresis analysis was carried out, and no degradation of the synthesized radiolabelled oligonucleotide was observed (Table 3).

Although the ^99m^Tc-GAGK-AMO-21 radiolabelling reaction has been appropriately optimized and the molecule’s stability has been verified in the serum environment, unfortunately, no hybridization studies or in vivo tests have been published yet to evaluate whether the two different chelating agents (GAGK vs. MAG3) actually confer different biological and pharmacokinetic behaviours to the probe.

### 5.2. ^68^Ga-Radiolabelled AMOs

^68^Ga is a positron-emitter radionuclide. It decays at 88.9% by emitting positrons with an average disintegration energy of 829.5 keV. This characteristic, combined with its relatively short half-life of approximately 68 min, makes ^68^Ga an excellent radioisotope for PET analysis, which is used both for diagnostic and imaging purposes. The widespread clinical use of ^68^Ga is facilitated by the availability of ^68^Ge/^68^Ga generators, which allow the radionuclide to be produced directly in nuclear medicine centres, without requiring a cyclotron. The generator eluate contains gallium in the form of ^68^GaCl_3_, with gallium in the +3 oxidation state. In aqueous solution, Ga^3+^ behaves as a hard Lewis acid and shows a marked tendency to form stable complexes with polydentate ligands. From a coordination chemistry perspective, Ga^3+^ exhibits a preference for octahedral geometries and binds effectively to macrocyclic chelators such as 1,4,7,10-tetraazacyclododecane-1,4,7,10-tetraacetic acid (DOTA) and 1,4,7-triazacyclononane-1,4,7-triacetic acid (NOTA) (Figure 6), which ensure high thermodynamic and kinetic stability of the complexes [65].

It is this characteristic that is exploited to bind ^68^Ga to carrier molecules such as proteins; antibodies; or, as in our case, oligonucleotides. Therefore, once the carrier molecule has been synthesized, two steps are necessary to obtain the complete radiotracer: the first involves the conjugation of the carrier molecule with the chelator, and the second is the actual radiolabelling [66,67].

This sequential process, while effective, requires additional time and manipulation before the radiotracer molecule is ready. However, recent studies have explored alternative strategies to simplify radiotracer preparation and improve overall efficiency.

Kiviniemi et al. [60] adopted this approach in their study published in 2012. They designed a method able to bind the chelator to the solid support that is used for the AMO synthesis. In this way, it is possible to proceed with a normal solid-phase synthesis of the oligonucleotide, which, once detached from the support, will only have to be radiolabelled to obtain the desired probe.

Starting from two commercially available precursors of DOTA and NOTA (Figure 7, **11** and **12**), the compounds shown in Figure 8 (**13**–**15**) were synthesized. Each of them displays a primary alcohol protected by a dimethoxytrityl (DMT) group, which constitutes a starting point for the synthesis of oligonucleotides. In particular, compound **14** contains the DOTA structure, while compounds **13** and **15** contain the NOTA structures. The complete synthetic procedures are reported in the cited publication [60].

To enable their use in solid-phase synthesis, these compounds must be linked to the support. The solid support used in this study was a porous glass with controlled porosity and derivatized with long-chain amines (LCAA-CPG). The synthesized compounds were anchored to the solid support through a succinic linker. For this purpose, compounds **13** and **14** were initially converted to the corresponding triol (**16**) and tetraol (**17**) through a transesterification reaction with 1,3-propandiol (Figure 9). These compounds were then treated with succinic anhydride, generating a mixture of mono-, di-, and tri-succinates. These can be easily linked to the solid support containing amino groups (LCAA-CPG) via an amide bond. At the end of the conjugation process, unreacted carboxylic acid groups were capped through amide bond formation using HBTU and benzylamine. Subsequently, any remaining hydroxyl and amino groups were blocked by acetylation with acetic anhydride. Only at this point were the functionalized solid supports (**18** and **19**, Figure 9) ready for use in oligonucleotide synthesis.

The same process was also followed for molecule **15** to obtain the modified solid support (**20**; Figure 10). First, **15** was converted into a mixture of mono- and di-succinates by reaction with succinic anhydride; then, it was linked to the solid support via the formation of an amide bond. Before being ready for use, the obtained solid support required capping of the carboxyl, amino and hydroxyl groups present on both the support and the chelator; these groups must be blocked to avoid side reactions during oligonucleotide synthesis.

The synthesis, developed by Kiviniemi et al. and described above, provides solid supports functionalized with three different chelators (compounds **18**, **19** and **20**). This strategy allows for the conjugation of the chelator to the 3′ end of the oligonucleotide, representing a particularly advantageous approach for diagnostic and therapeutic applications based on functionalized oligonucleotides. A particularly relevant aspect of this strategy is the possibility of keeping the 5′ terminus free, which can subsequently be used for the introduction of further biomolecules or functional units, such as peptides, aptamers or fluorophores. Furthermore, the integration of the chelator during the solid-phase synthesis of the oligonucleotide allows the main limitations associated with post-synthetic conjugations, including the heterogeneity of the final product, low conjugation yields, and difficulties in purification processes, to be overcome.

To verify the validity of the developed system, three miR-15b antisense oligonucleotides (AMO-15b) were synthesized (compounds **21**, **22** and **23**; Figure 11) by the Kiviniemi group, each using one of the functionalized solid supports (**18**, **19**, or **20**).

It is known that miR-15b plays a role in bone remodelling. Specifically, miR-15b is involved in osteoblast differentiation and bone formation [68]. The choice of this miRNA as a target allowed for a detailed analysis of the specificity of the accumulation of synthesized AMO-15b tracers in bone tissue.

The three AMO-15bs (22 mer long, with the same base sequence but each having a different chelator) were synthesized using all 2′OMe nucleotides. Subsequently, compounds **21**, **22**, and **23** were radiolabelled with ^68^Ga. Gallium was obtained by elution of a ^68^Ge/^68^Ga generator using 0.1 M HCl. Sodium acetate was added to 1 mL of the resulting eluate until a pH of 5.5 was reached. Conjugate **21** was then added to this solution, and the reaction mixture was heated at 100 °C for 10 min. After the reaction, the mixture was purified by solid-phase extraction (SPE) using a C18 column. The same protocol was also applied to compounds **22** and **23**.

The biodistribution of these three radiolabelled AMOs in rats was then evaluated. No significant differences were observed in the biodistribution of the three miR-15b antisense oligonucleotides, indicating that the variation of the chelating agent does not affect target affinity or specificity.

Then, the biodistribution data were compared with those of the T6 oligodeoxyribonucleotide. T6 is an oligonucleotide composed of six thymines used as a control. T6 is a short, simple, and stable sequence, perfect for verifying that the new conjugation and radiolabelling method works correctly before moving on to biologically relevant sequences such as AMO-15b. Indeed, in this case too, T6 was conjugated with the three bifunctional chelating agents, then labelled with ^68^Ga.

Marked differences emerged when comparing the biodistribution of the three radiolabelled AMO-15bs with those of the corresponding radiolabelled T6 oligonucleotides. The radiolabelled AMO-15bs showed greater radioactive accumulation in the kidneys, bones, and bone marrow than T6 oligonucleotides. The latter were mainly excreted via urine and showed only modest accumulation in the small intestine and white adipose tissue. This difference in biodistribution, particularly the bone accumulation observed for radiolabelled AMO-15b, regardless of the chelator used, indicates that the sequence complementary to the target is a key determinant for effective target localization.

The same research group extended the biodistribution studies in rats in a subsequent work [61]; the three AMO-15bs bearing the three different chelators (Figure 11, compounds **21**, **22** and **23**) were resynthesized and labelled with ^68^Ga.

In this case, however, the radiolabelling conditions were changed. ^68^Ga was obtained as ^68^GaCl_3_ by elution of a ^68^Ge/^68^Ga generator with 0.1 M HCl. Here, the eluate was buffered with sodium acetate to a final concentration of 0.4 M, and the pH was adjusted to 3.5. Subsequently, the conjugated oligonucleotide (5–12 nmol) was added, and the reaction mixture was heated to 95 °C for 10–15 min.

Again, the three radiolabelled AMO-15bs showed very similar biodistributions, confirming that the chelators do not significantly affect the biodistribution or behaviour of the antisense oligonucleotides.

To conduct a more comprehensive study, a scramble of the AMO-15b and an AMO-15b with only two modified bases (AMO-15b-2SBS) were also synthesized using the functionalized solid support (**18**). The scramble contains the same nucleotides as AMO-15b but in a different order so that it has no target in the rat genome, while AMO-15b-2SBS has the same nucleotides sequence as AMO-15b but at the 3′ end, a guanine has been changed to an adenine and a uracil to a guanine. Biodistribution tests in rats were also performed on the bifunctional NOTA chelator alone (compound **10**) and on the T6 oligonucleotide synthesized using the same functionalized solid support (**18**). All synthesized conjugates (AMO-15b-2SBS, AMO-15b scramble, NOTA, and T6-NOTA) were radiolabelled with ^68^Ga using the same procedure described above.

Biodistribution studies in rats showed that radiolabelled AMO-15b, AMO-15b-2SBS, and the AMO-15b scramble accumulated primarily in the kidneys, liver, and urine. Accumulation was also observed in the epiphyses of long bones, although in varying amounts: greater for AMO-15b, slightly less for AMO-15b-2SBS, and even lower for the scramble. The biodistribution of NOTA and T6-NOTA was evaluated too. The latter two showed similar biodistributions, differing from those of the other oligonucleotides. In fact, they do not accumulate in the bones, but a greater presence in the intestine and in the urine was found. These biodistribution data indicate that the radiolabelled probe accumulates more efficiently at the target site when using AMO-15b, whose sequence is fully complementary to the miRNA of interest. This finding is consistent with sequence-dependent biodistribution, although it does not, by itself, demonstrate direct intracellular miRNA binding.

## 6. Radiolabelled AMOs for Therapeutic Purposes

At present, several radionuclides, including β^−^-emitting isotopes such as ^177^Lu and ^131^I, are widely used in clinically approved radiopharmaceuticals for therapeutic applications [69]. In this context, the therapeutic application of radiolabelled AMOs remains at an early preclinical stage.

Preliminary studies have begun to explore this possibility. In particular, Xian et al. [70] reported the development of a ^131^I-labelled AMO-21 for theranostic applications in prostate tumour xenografts. In this approach, a tyrosine residue was conjugated to the 3′ end of the AMO through a C6 linker, enabling radioiodination with ^131^I (radiochemical yield of 95.5% ± 3.1, *n* = 3). ^131^I is a β^−^- and γ-emitting radionuclide; therefore, it combines therapeutic potential with the possibility of monitoring the biodistribution of the treatment in vivo by SPECT. ^131^I-AMO-21 synthesized in this study showed high radiochemical stability, and after its IV administration to tumour-bearing mice, the tumour region was clearly shown by SPECT images in one of the models used (DU145 xenograft). Its therapeutic activity was demonstrated in vitro through inhibition of cell growth and induction of apoptosis. These findings further support the potential of these molecules as a promising tool for the development of innovative radiopharmaceuticals.

While radioiodination represents one possible strategy for the therapeutic radiolabelling of AMOs, an alternative approach could build on the radiolabelling strategies already established for diagnostic applications. To date, AMOs have been conjugated to β^+^- or γ-emitting radioisotopes using bifunctional chelating agents such as DOTA, NOTA, and MAG3. Similar to other approved radiopharmaceuticals, once the oligonucleotide is functionalized with a suitable bifunctional chelator, the resulting construct could potentially serve as a versatile platform for the coordination of different radionuclides. This strategy may enable the modulation of the biological application of the same molecular scaffold, allowing for a shift from diagnostic to therapeutic purposes depending on the radionuclide employed. However, changing the coordinated radionuclide would require a reassessment of the radioconjugate’s stability, dosimetry, and toxicity, as these parameters may vary depending on the physicochemical and radiobiological properties of the selected radionuclide. A representative example is DOTA-TATE, a somatostatin analogue functionalized with macrocyclic chelator DOTA, whose clinical use varies according to the coordinated radioisotope: ^68^Ga for positron emission tomography imaging or ^177^Lu for targeted radionuclide therapy [71].

However, before fully exploring therapeutic applications, it is essential to optimize diagnostic approaches. γ- or β^+^-emitting radioisotopes are more easily detectable and monitored, allowing for accurate traceability of molecules both in vitro and in vivo. For this reason, the development of diagnostic radiopharmaceuticals generally precedes that of the corresponding therapeutic agents. In the specific case of radiolabelled AMOs, significant challenges remain, particularly regarding delivery efficiency and biodistribution, aspects crucial to the success of future clinical applications.

## 7. Delivery Strategies for Radiolabelled AMOs

AMOs are relatively long, polyanionic and highly hydrophilic molecules. These characteristics limit their ability to cross the cell membrane by passive diffusion, representing a major challenge for their cellular delivery. However, as previously reported, initial in vitro and in vivo studies suggest that a fraction of radiolabelled AMOs modified with 2′OMe and PS bonds are capable of entering cells, even in the absence of dedicated drug delivery systems. Despite this, the uptake mechanism requires further investigation and optimization.

Over the years, new strategies have been developed for oligonucleotide delivery, whether single-stranded antisense oligonucleotides (ASOs), antisense miRNA oligonucleotides (AMOs), or double-stranded small interfering RNAs (siRNAs).

Several delivery systems have been proposed to date. Among these, bioconjugation-based systems are among the main approaches obtained by attaching a more lipophilic moiety to one end of the oligonucleotide [72]. This lipophilic component can consist of a peptide sequence [73], a lipid group [74], or a sugar molecule (e.g., N-acetylgalactosamine) [75], with the aim of improving interaction with cell membranes and facilitating cell uptake. Conjugates are often designed to disassemble after cell entry through the use of acid-labile linkers, which break down in the endosomal microenvironment. Furthermore, the interaction between the lipophilic component and specific cellular receptors involved in endocytosis processes can help increase the specificity of the delivery system [76].

Another widely studied strategy involves the use of encapsulation systems in which the oligonucleotide is enclosed within nanoparticles or vesicles. Nanoparticles offer the advantage of being able to be designed by modulating various biophysical and biological properties, such as shape, size, composition, and surface functionalization. This high versatility allows these systems to be optimized according to the specific therapeutic or experimental objective. However, they can present problems of immunogenicity and rapid clearance by the reticuloendothelial system [77]. Endogenous vesicles [78] such as exosomes, on the other hand, represent a natural and biocompatible delivery system, with reduced immunogenicity and the intrinsic ability to cross biological barriers such as the blood–brain barrier. However, large-scale production, efficient loading, and targeted delivery remain significant challenges for their clinical application.

Regarding radiolabelled AMOs, the delivery systems currently being explored are still limited. Although their characteristics are similar to those of other oligonucleotides, the presence of the radioactive component introduces specific constraints in terms of timing and applicable strategies, which are not found in conventional systems.

A method for improving radiolabelled AMO delivery was recently investigated by the Peking University group led by Kang. The authors developed a system based on lipid nanoparticles (NPs) conjugated with the previously studied ^99^^m^Tc-MAG3-AMO-155 [58]. The formation of ^99^^m^Tc-AMO-NP conjugates occurs primarily through electrostatic interactions between the negatively charged AMOs and the positively charged liposome surface. The resulting conjugates were characterized in terms of serum stability and cellular uptake, showing significantly superior performance compared to free AMO. Furthermore, the efficacy of in vivo imaging was evaluated using a cervical cancer model. Biodistribution studies revealed significantly greater accumulation of the ^99^^m^Tc-AMO-NP complex at the tumour level compared to the control (2.7 ± 0.6% ID/g vs. 0.4 ± 0.1% ID/g). Furthermore, the nanoparticle system showed a longer circulating half-life and reduced renal elimination, although hepatic accumulation was observed, consistent with the typical behaviour of lipid nanocarriers [79].

An alternative strategy to enhance AMO delivery involves the use of cell-penetrating peptides (CPPs), a class of peptides capable of promoting the intracellular transport of nucleic acids and other bioactive molecules. Positively charged CPPs can form non-covalent complexes with AMOs, promoting their cell penetration and enhancing tumour signalling. In this context, Yang et al. developed a conjugate consisting of AMO-21 and the PepFect6 peptide (PF6). AMO-21 is a 22-mer single-stranded oligonucleotide complementary to miR-21. To increase its stability, the oligonucleotide was modified at both ends by introducing three PSs and three 2′OMe nucleotides on each side. The oligonucleotide was subsequently labelled with ^99^^m^Tc using hydrazinonicotinamide (HYNIC) as a bifunctional chelating agent. To enhance delivery, the PepFect6 (PF6) peptide, derived from CPP transporter 10 (TP10), was engineered by introducing stearyl and trifluoromethylquinoline modifications to improve transfection efficiency and facilitate endosomal escape of the oligonucleotide. The efficacy of the ^99^^m^Tc-AMO-21/PF6 system (average diameter of 30–40 nm) was evaluated in a xenograft model of A549 lung adenocarcinoma, comparing it to Lipofectamine 2000-based nanoparticles (^99^^m^Tc-AMO-21/LIP, average diameter of 50–70 nm).

Results showed significantly higher cellular uptake (11.24 ± 0.12 vs. 8.66 ± 0.20 mol/cell at 12h) and retention (3.92 ± 0.15 vs. 2.12 ± 0.10 mol/cell at 12h) for ^99^^m^Tc-AMO/PF6 compared to ^99^^m^Tc-AMO-21/LIP. In vivo, the ^99^^m^Tc-AMO/PF6 nanoprobe provided a superior tumour-to-muscle (T/M) ratio at 6 h post injection (21.76 ± 0.98 vs. 11.77 ± 2.60) and an enhanced tumour-to-liver ratio (1.46 ± 0.05 vs. 1.32 ± 0.05), thereby exhibiting a higher overall target-to-background contrast compared to the Lipofectamine formulation. Despite the promising results, the system requires further optimization to improve its stability and imaging efficiency, as well as further in vitro and in vivo validation on different tumour models [80].

## 8. Conclusions

This overview describes how, among the various critical factors for the development of radiolabelled AMOs, stability of the molecule both at the high temperatures required during the radiolabelling phase and when administered in vivo can represent a significant area of concern. Fortunately, this critical issue can be effectively overcome through appropriate chemical modifications at both the backbone and sugar levels, giving the oligonucleotides adequate chemical and biological robustness.

The conjugation of oligonucleotides and radioisotopes, mediated by bifunctional chelating agents, represents an effective strategy for rapid radiolabelling with high radiochemical yields. Furthermore, the use of a single chelating agent capable of complexing different radioisotopes could allow for modulation of the radiopharmaceutical’s clinical application, expanding its potential versatility from diagnostic to therapeutic settings. However, radiochemical stability and high radiolabelling yields alone are not sufficient to ensure the effectiveness of these systems as imaging agents. Biodistribution; tumour uptake; clearance; and, in particular, the T/NT ratio remain crucial aspects that must be carefully considered when evaluating their potential for in vivo applications.

Regarding cellular delivery, preliminary studies indicate that structural modifications of oligonucleotides enable partial cellular uptake; however, the extent of internalization remains insufficient to make these systems competitive as diagnostic tools. In recent years, increasing attention has been directed toward the use of drug delivery strategies, such as nanoparticles and cell-penetrating peptides, which have shown promising results, although further optimization and validation are still required.

An additional aspect emerging from the analysis of the different chemical modification strategies is the limited use of LNA chemistry in radiolabelled AMOs. LNA modifications are widely employed in the development of therapeutic oligonucleotides because of their high affinity for complementary sequences and have already reached clinical evaluation in antisense miRNA therapeutics. Examples are miravirsen, targeting miR-122, and CDR132L, targeting miR-132 [81,82]. Despite this clinical experience, the potential of LNA-modified AMOs as molecular imaging probes remains largely unexplored. Therefore, it would be of interest to investigate whether the incorporation of LNA into AMOs already developed for the imaging of miRNAs, including those targeting miR-21 and miR-155, could favourably influence properties such as molecular stability, target affinity and biodistribution. Direct comparison of different chemistries using the same AMO could help determine whether the benefits of LNA observed in the therapeutic setting with miravirsen and CDR132L can also be applied to radiolabelled AMOs for molecular imaging.

The available studies have also highlighted an additional limitation that should be considered when selecting the radionuclide for imaging. In particular, the relatively slow pharmacokinetics of oligonucleotide-based probes may represent a significant challenge for PET imaging. The studies performed with ^99m^Tc showed that tumour-associated uptake and tumour/non-tumour ratios may require several hours to reach favourable values. Although this behaviour may be advantageous for target retention, it can become problematic when considering short-lived PET radionuclides such as ^68^Ga. A biological process requiring several hours to reach optimal tumour-to-background contrast is poorly matched with the physical half-life of ^68^Ga, potentially resulting in a substantial loss of radioactive signal before the most informative imaging time point is reached. Therefore, the biological kinetics of the probe should be considered together with the physical properties of the radionuclide from the earliest stages of tracer design.

In addition, the interpretation of tumour uptake remains challenging because accumulation of the radiolabel does not necessarily reflect specific interaction with the target miRNA. Renal and hepatic accumulation, as well as differences in clearance pathways, can significantly influence the observed signal and, consequently, the tumour-to-background contrast. This nonspecific accumulation may be particularly problematic for tumours located in the abdominal region, where increased background activity can hinder lesion visualization. For this reason, appropriate controls, including scrambled or sequence-modified probes, together with complementary molecular techniques, are essential to demonstrate that the observed signal is actually associated with target recognition. This aspect is particularly important, considering the multifunctional role of miRNAs, whose expression alone may not always provide an unambiguous indication of a specific pathological state.

In this context, it is also important to consider that direct targeting of miRNAs with radiolabelled AMOs is not the only possible strategy. As discussed by Zientek et al. [83], miRNAs may be approached through different molecular strategies, including small molecules capable of directly interacting with the miRNA sequence; molecules targeting proteins involved in miRNA function, such as AGO2; and direct targeting through antisense oligonucleotides. Small molecules may be particularly attractive for PET imaging because their smaller size can favour tissue penetration and more rapid pharmacokinetics compared with oligonucleotide-based probes. These properties may facilitate faster biodistribution and access to tissues that are more difficult to reach with larger oligonucleotide-based probes, including those protected by biological barriers. Small-molecule approaches targeting miR-21, for example, have already been investigated, demonstrating the possibility of combining miRNA recognition with radiochemistry suitable for PET. At the same time, indirect targeting of miRNA-associated proteins represents another potentially interesting approach, since targeting the molecular machinery involved in miRNA function could overcome some of the limitations associated with direct recognition of the RNA sequence. These alternative strategies are still not fully developed, and further work will be necessary to establish their specificity and their suitability for in vivo imaging. Nevertheless, they underline that the development of miRNA imaging agents should not necessarily be restricted to the AMO approach but should, instead, consider the biological characteristics of each target and the pharmacokinetic requirements imposed by the selected imaging modality.

An additional consideration emerging from the literature is the limited number of recent studies specifically focused on radiolabelled AMOs. Although new contributions have been published, mainly addressing the delivery and in vivo distribution of these probes [79], the overall body of evidence remains relatively small. This trend suggests that radiolabelling of AMOs still represents a niche research area in which several fundamental challenges remain unresolved. In particular, the need to combine adequate probe stability with efficient biodistribution, sufficient intracellular access and convincing demonstration of target specificity may have limited the broader development of this approach. Further advances in chemical modification and delivery strategies could therefore play an important role in promoting the evolution of radiolabelled AMOs as molecular imaging tools.

Overall, the studies discussed in this review demonstrate the potential of radiolabelled AMOs for the imaging of miRNAs while also showing that several challenges still need to be overcome before their translation into clinically useful imaging agents. Future studies should therefore focus not only on improving radiolabelling efficiency and stability but also on optimizing delivery, intracellular accessibility, target specificity and pharmacokinetic behaviour. In parallel, the development of alternative targeting strategies, particularly small-molecule approaches, may provide complementary solutions and could ultimately help to overcome some of the limitations currently associated with oligonucleotide-based probes. Further investigation of these different approaches, supported by rigorous in vivo validation and complementary molecular analyses, will be essential to determine the real potential of miRNA imaging in cancer and other diseases.

## Figures and Tables

**Figure 1:**
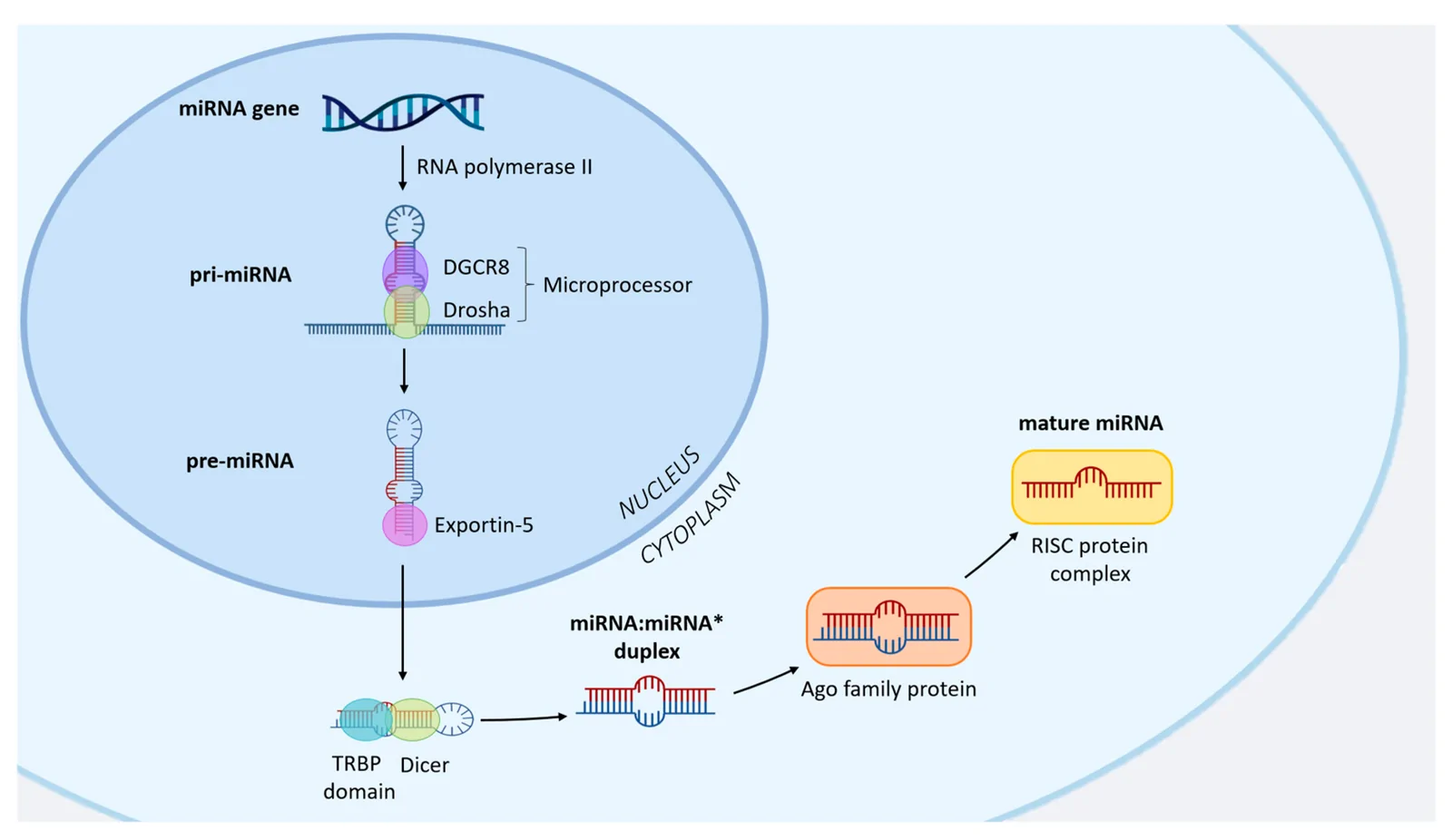
Schematic representation of the canonical miRNA biogenesis pathway. Pri-miRNAs are processed by the Drosha–DGCR8 complex into pre-miRNAs, exported from the nucleus by Exportin-5, and cleaved by Dicer to generate a miRNA duplex. The guide strand is subsequently loaded into the Argonaute (Ago)-containing RNA-induced silencing complex (RISC), forming the mature miRNA responsible for post-transcriptional gene regulation.

**Figure 2:**
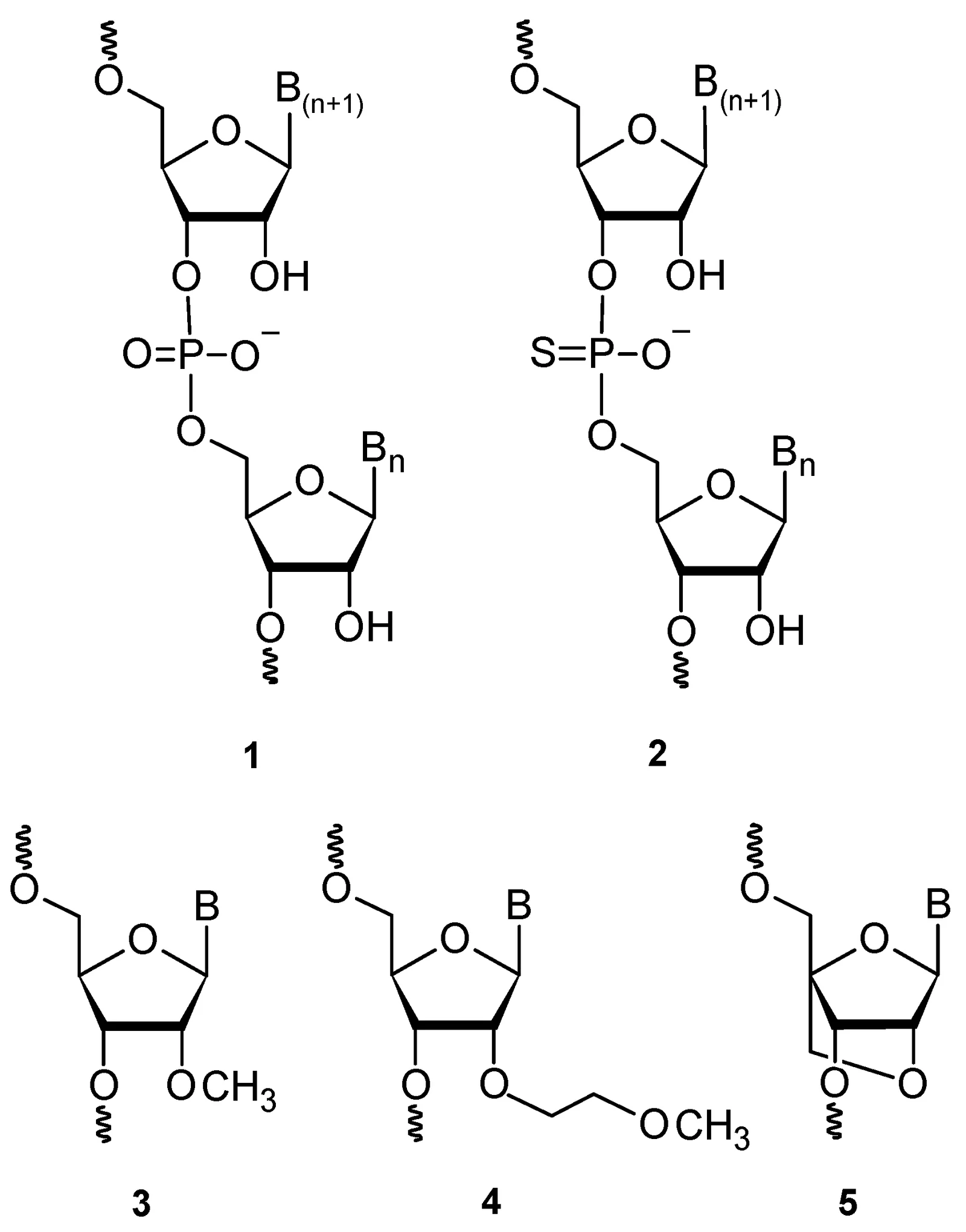
PO (**1**) and PS (**2**). Sugar modifications on the 2′ position: 2′OMe (**3**), 2′MOE (**4**), and LNA (**5**).

**Figure 3:**
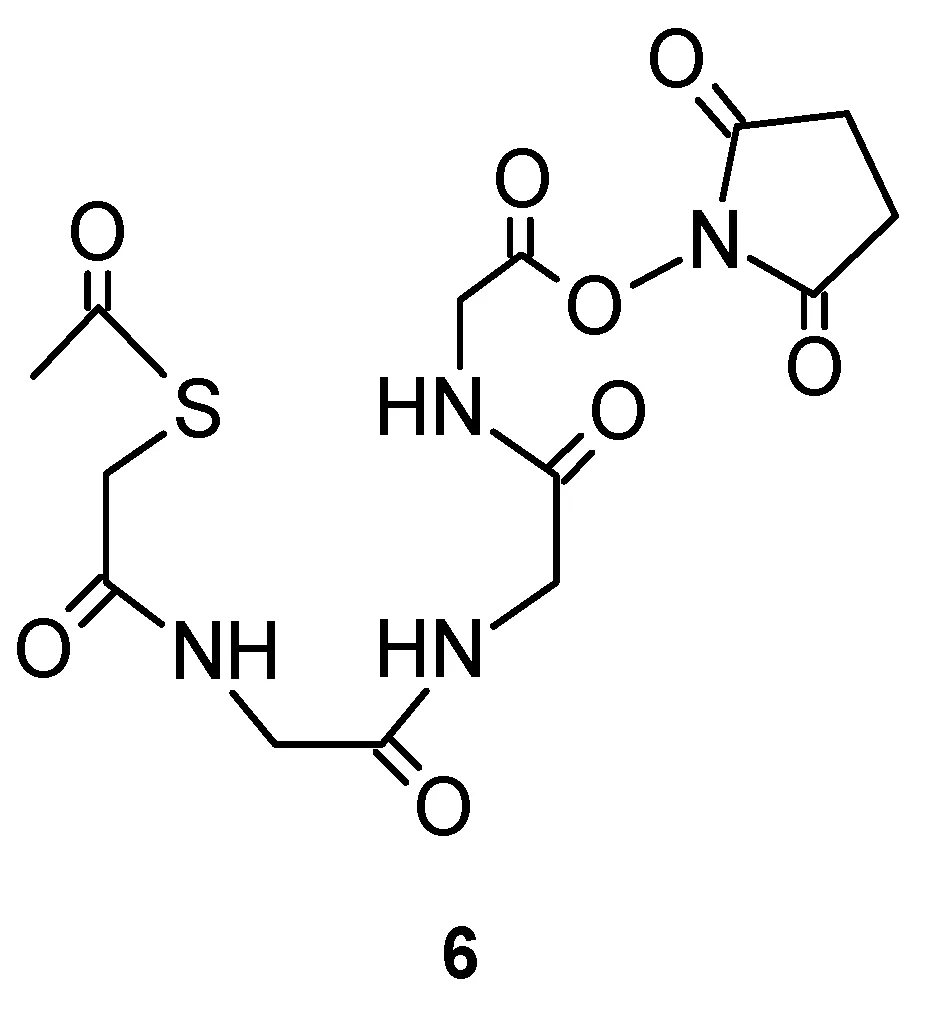
*S*-Acetyl NHS-MAG3 (*N*-hydroxysuccinimidyl derivative of *S*-acetylmercaptoacetyltriglycine) (**6**).

**Figure 4:**
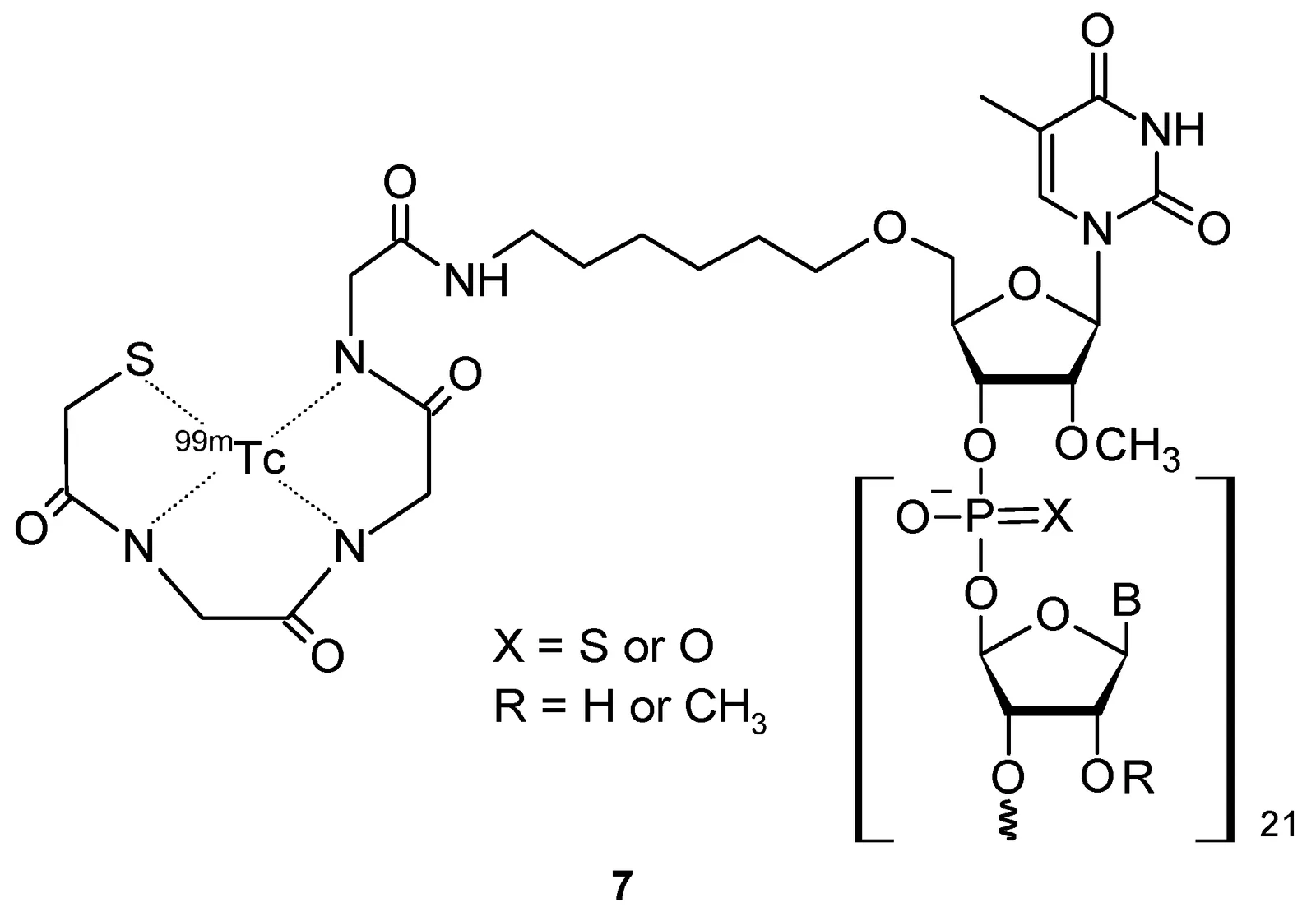
^99m^Tc-MAG3-AMO-21 (**7**) [54].

**Figure 5:**
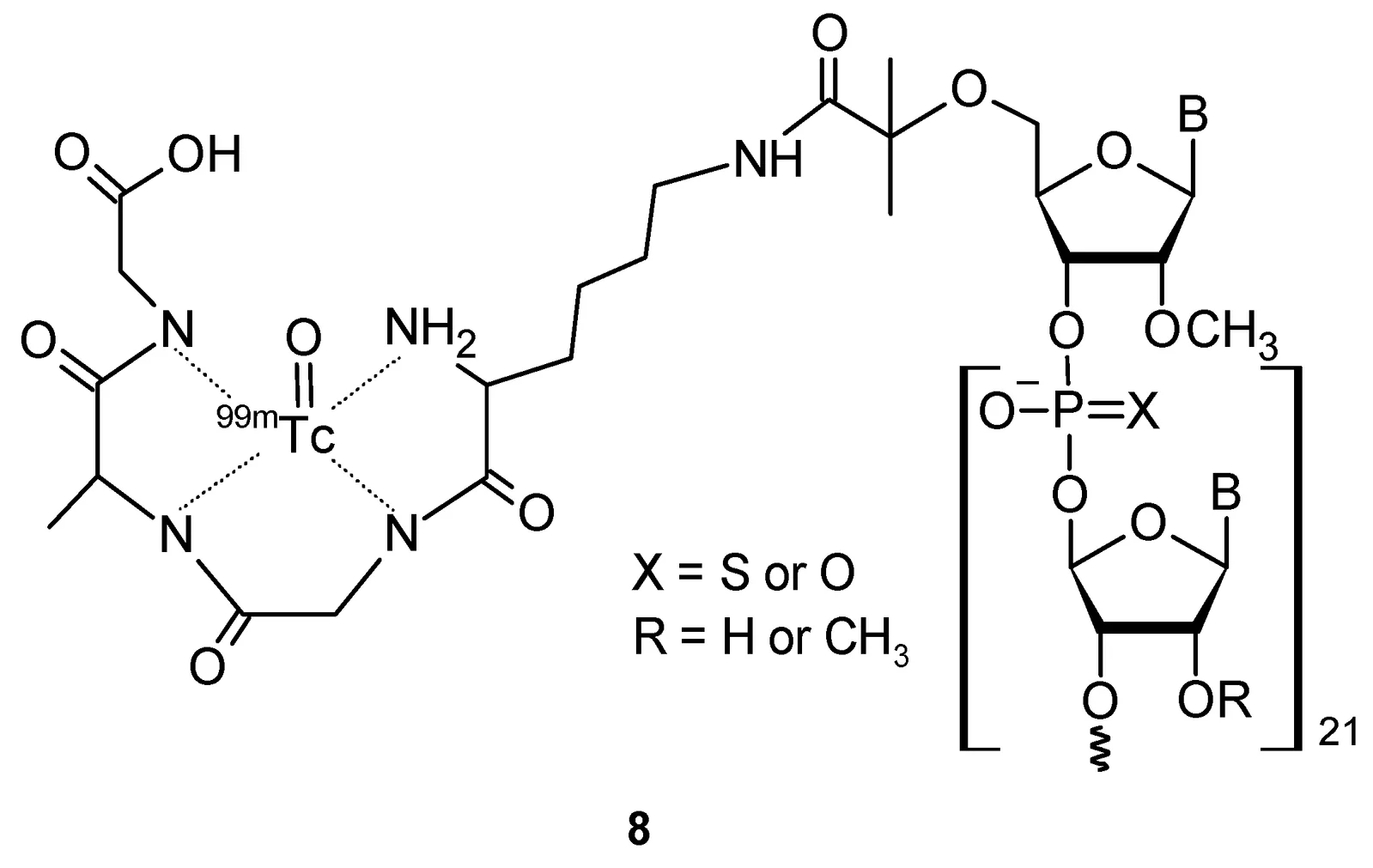
^99m^Tc-GAGK-AMO-21 (**8**) [59].

**Figure 6:**
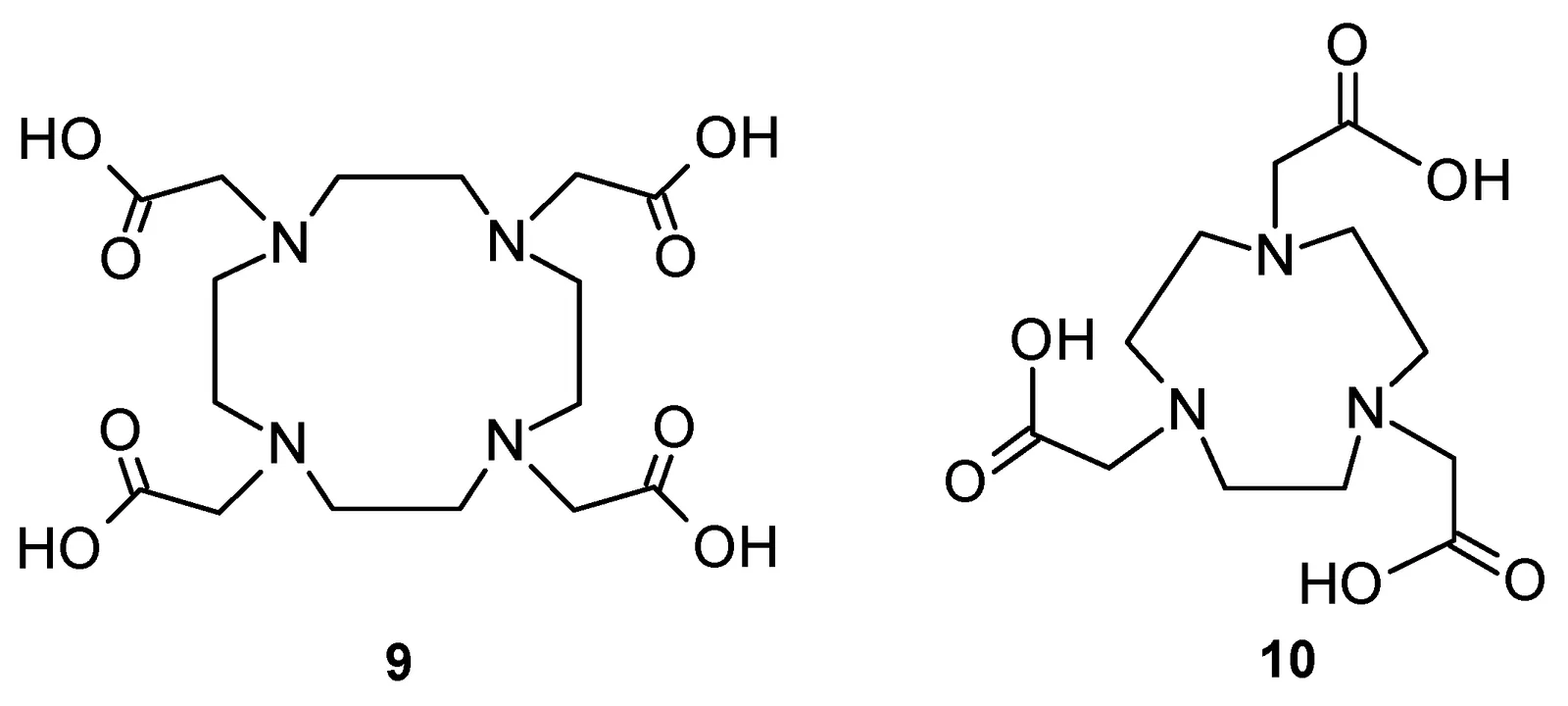
DOTA (**9**) and NOTA (**10**).

**Figure 7:**
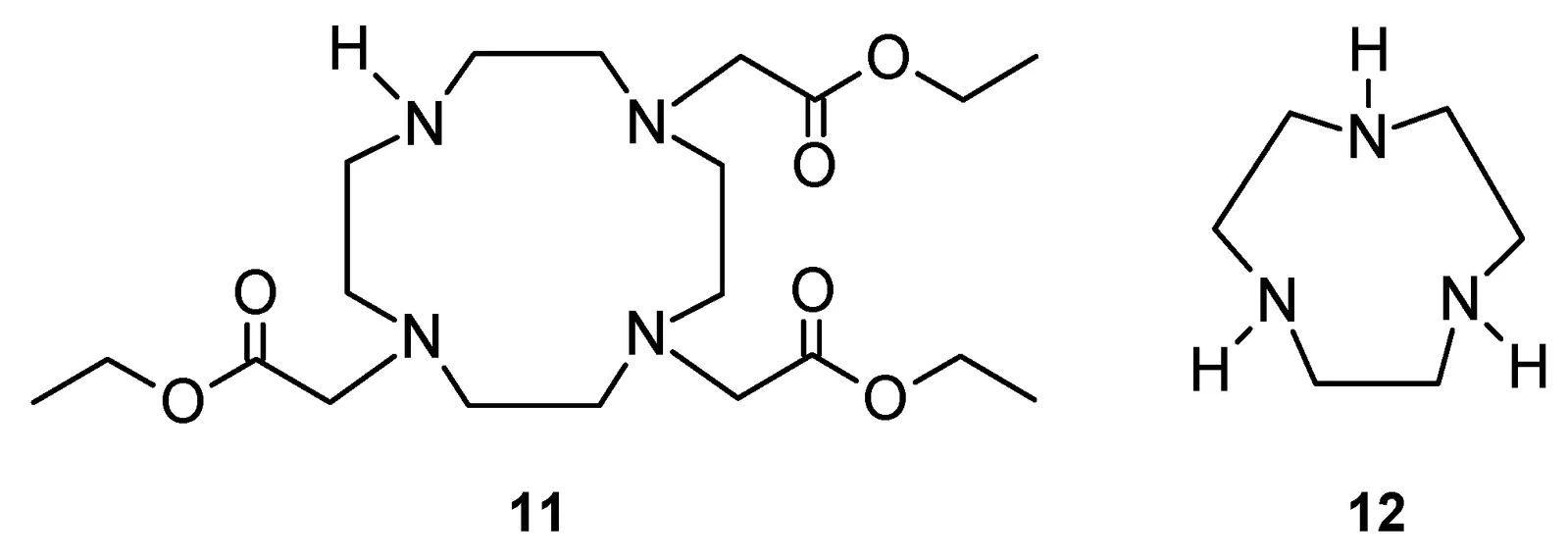
Triethyl 2,2′,2″-(1,4,7,10-tetraazacyclododecane-1,4,7,-triyl)triacetate (**11**) and 1,4,7-triazacyclononane (**12**).

**Figure 8:**
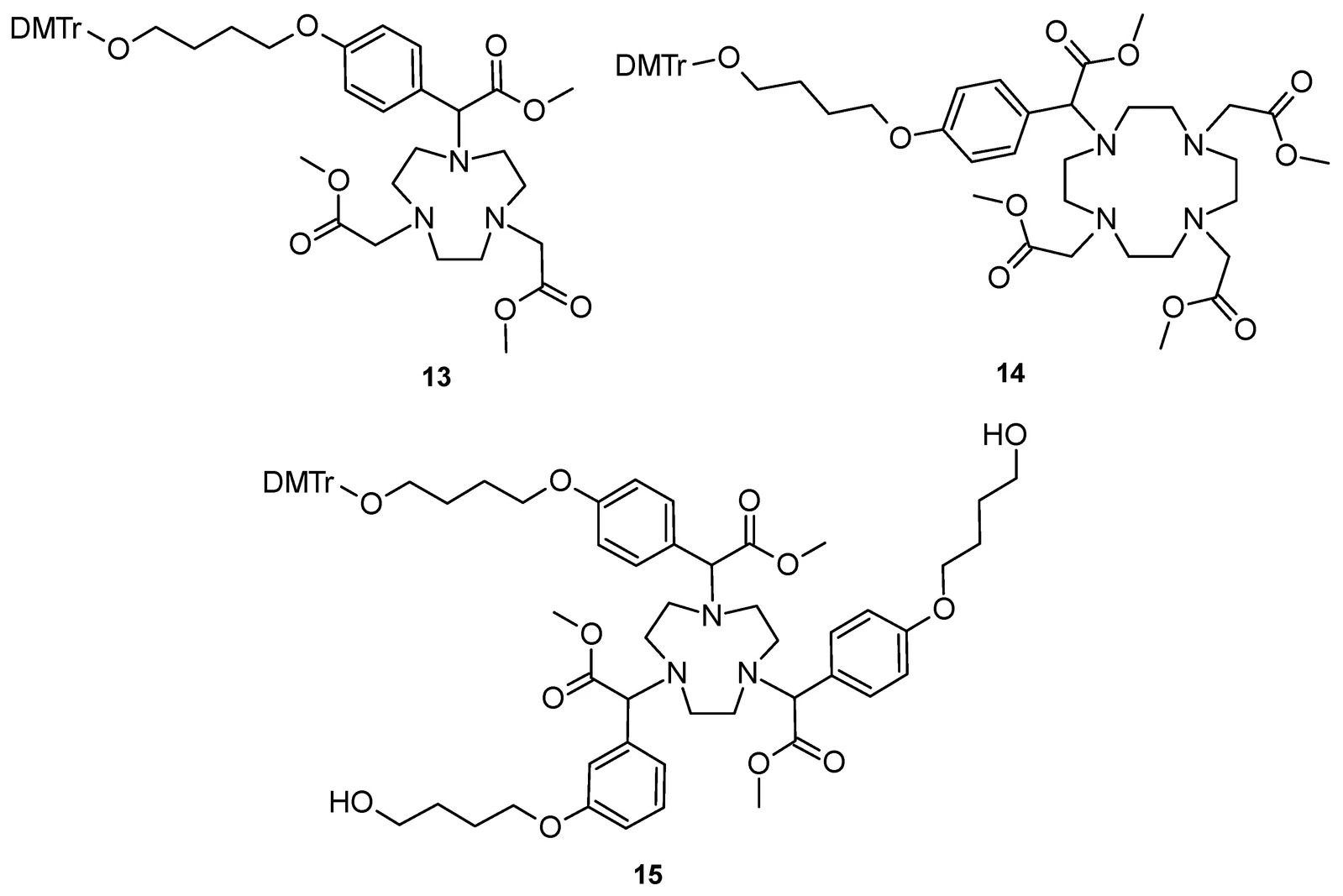
(**13**) Dimethyl [7-(1-{4-[4-(4,4′-Dimethoxytrityloxy)-butoxy]phenyl}-2-methoxy-2-oxoethyl)-1,4,7-triazacyclononane-1,4-diyl]diacetate; (**14**) trimethyl 2,2′,2″-[10-(1-{4-[4-(4,4′-dimethoxytrityloxy)-butoxy]phenyl}-2-methoxy-2-oxoethyl)-1,4,7,10-tetraazacyclododecane-1,4,7-triyl]triacetate; (**15**) dimethyl [7-(1-{4-[4-(4,4′-dimethoxytrityloxy)-butoxy]phenyl}-2-methoxy-2-oxoethyl)-1,4,7-triazacyclononane-1,4-diyl]bis{2-[4-(4-hydroxybutoxy)phenyl]-acetate}.

**Figure 9:**
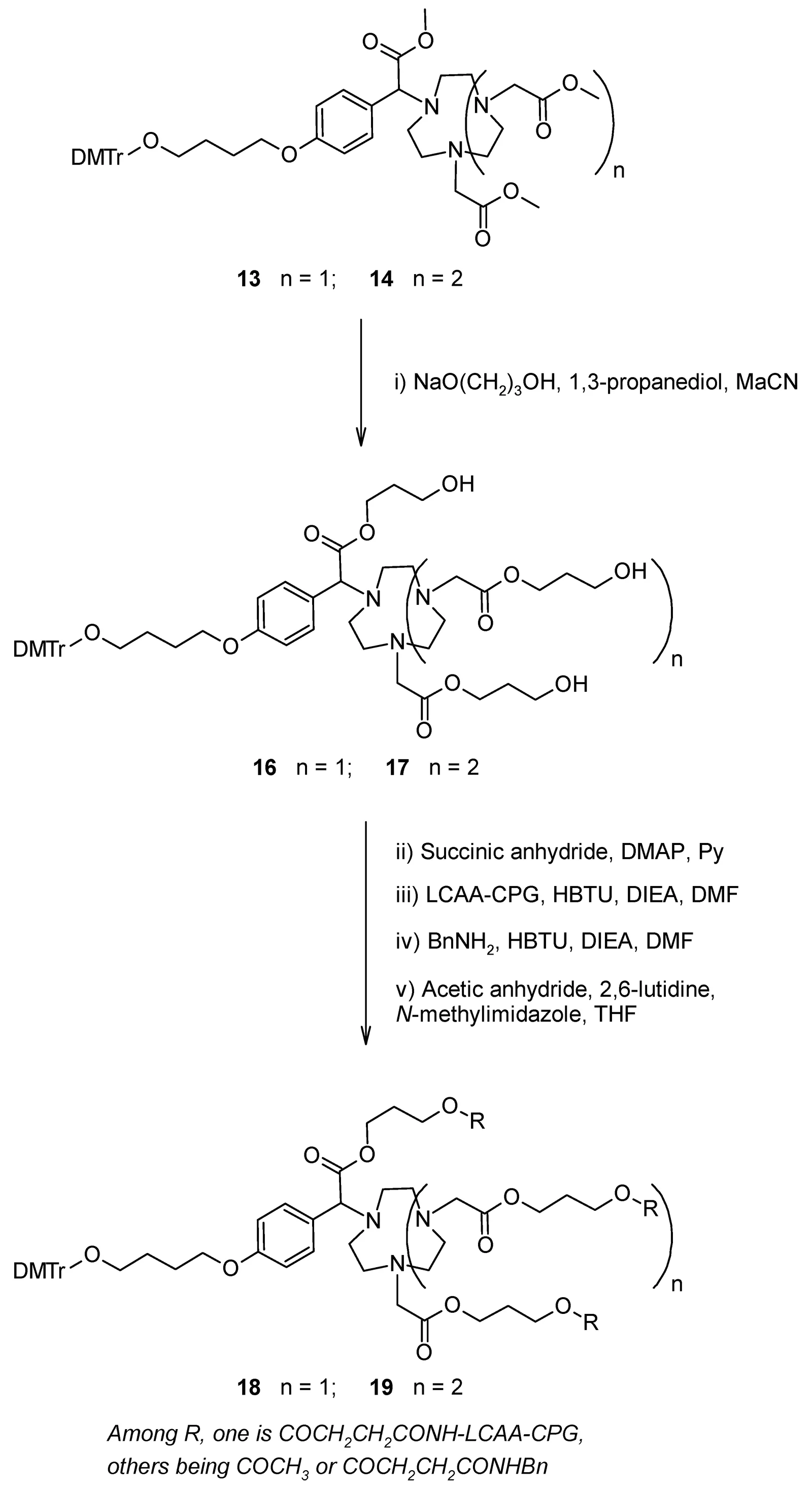
Synthesis of functionalized solid supports **18** and **19** starting from compounds **13** and **14**.

**Figure 10:**
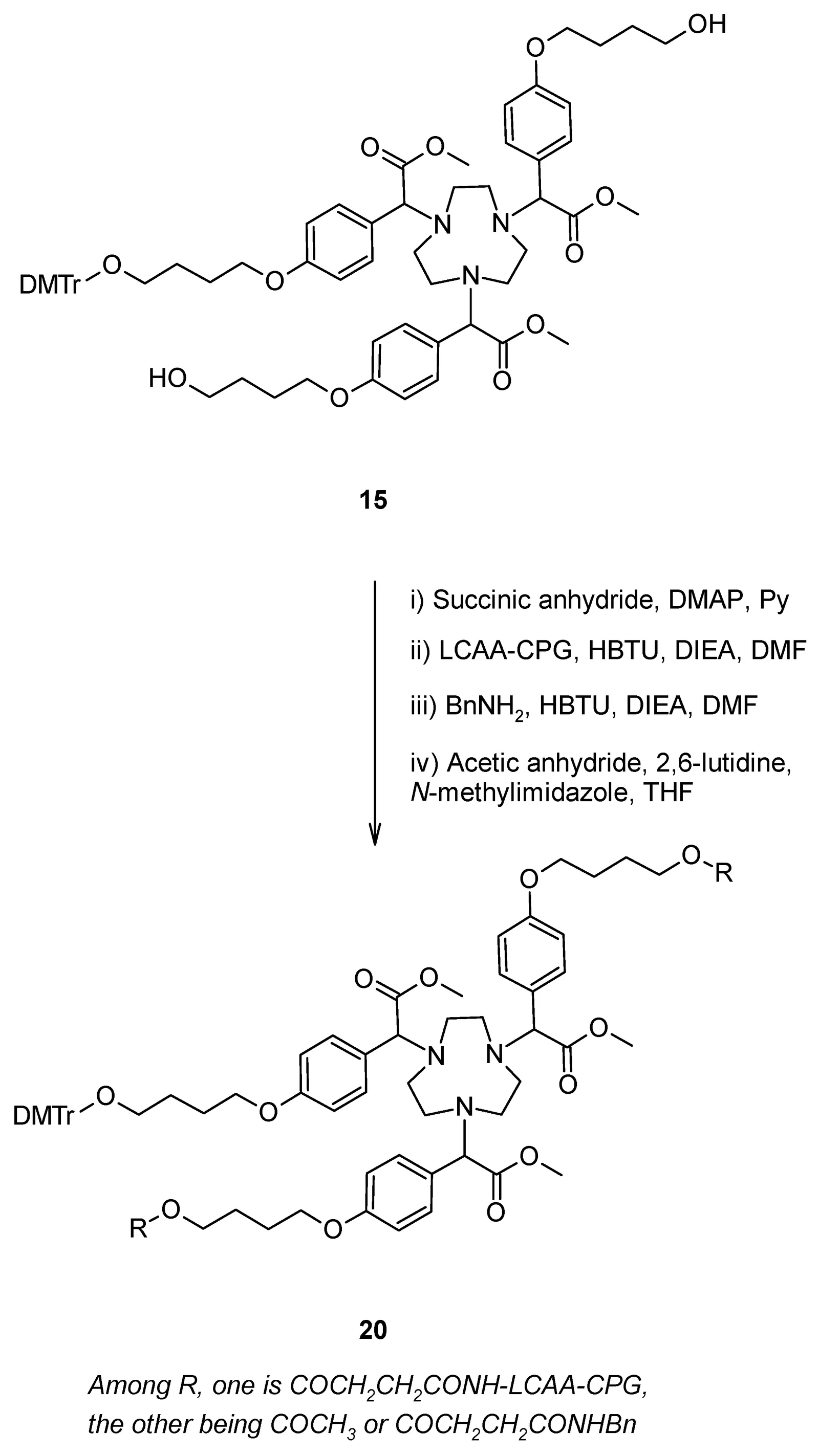
Synthesis of functionalized solid support **20** starting from compound **15**.

**Figure 11:**
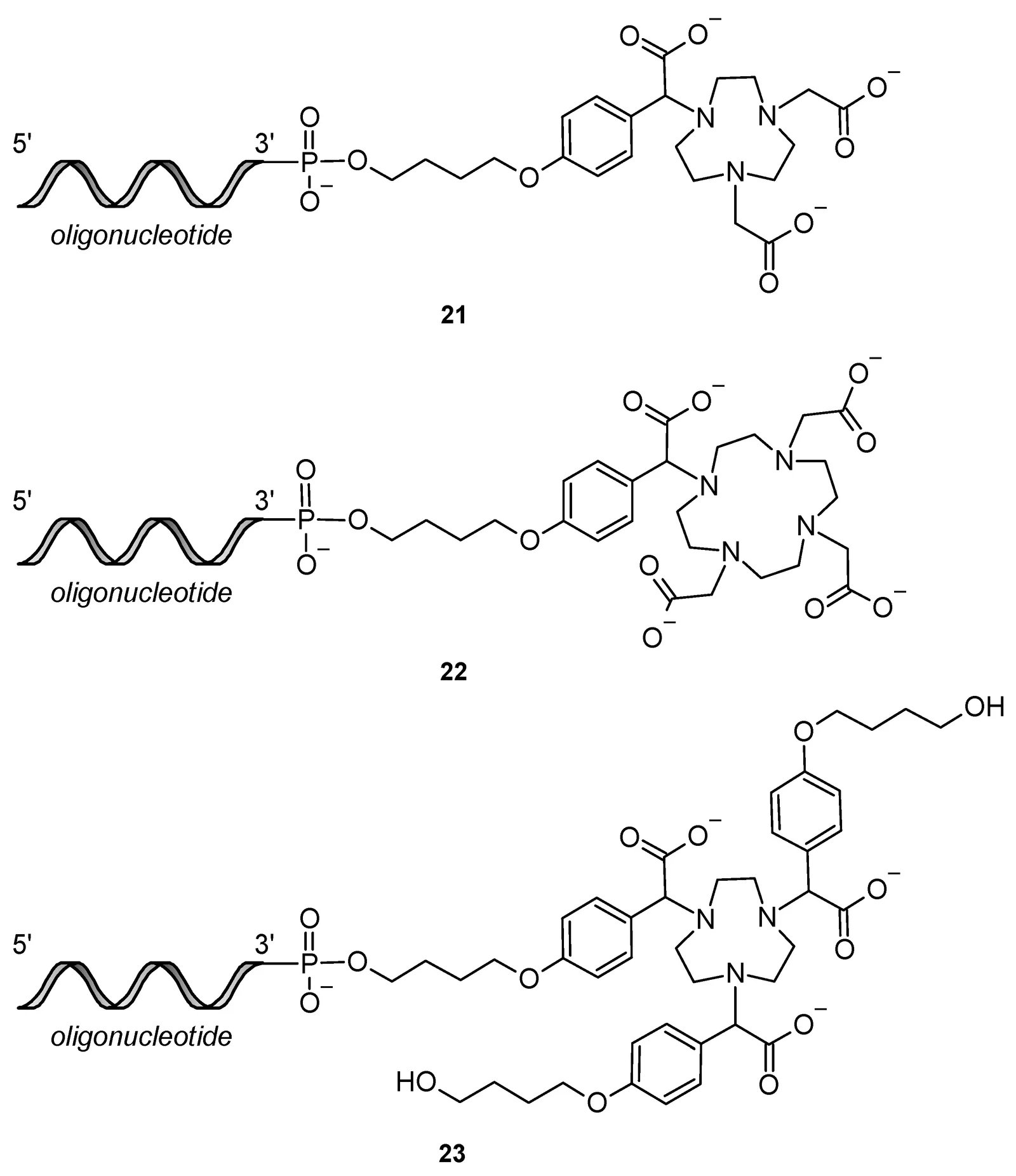
Oligonucleotides conjugated to three bifunctional chelating agents: NOTA (**21**); DOTA (**22**); NOTA-modified (**23**).

**Table 2:** Comparison between ^99m^Tc-MAG3-AMO-21 and ^99m^Tc-MAG3-AMO-155 tumour uptake and T/B ratios.

	^99m^Tc-MAG3-AMO-21	^99m^Tc-MAG3-AMO-155
**In vivo** **models**	HeLa tumor-bearing mice (*n* = 3 for each timepoint)	MCF-7 tumor-bearing mice (*n* = 3 for each timepoint)
**Timepoints**	0.5, 1, 2, 4, 6, 8 h	1, 2, 4, 6, 8 h
**Tumor up-take** **(% ID/g)**	2.11 ± 0.42 (0.5 h) to 0.91 ± 0.07 (8 h)	5.17 ± 1.07 (1 h) to 3.72 ± 0.43 (8 h)
**Tumor/Blood ratio**	2.52 ± 0.56 (8 h)	4.74 ± 0.59 (8 h)

**Table 3:** Overview of ^99m^Tc-radiolabelled AMO studies.

Radiolabelled AMO	In Vitro Serum Stability (Gel Electrophoresis)	In Vitro and In Vivo Studies	References
**^99m^** **Tc-MAG3-AMO-21**	Stable up to 12 h	yes	[54,57]
**^99m^** **Tc-MAG3-AMO-155**	Stable up to 12 h	Yes	[58]
**^99m^** **Tc-GAGK-AMO-21**	Stable up to 12 h	No	[59]

## Data Availability

No new data were created or analysed in this study. Data sharing is not applicable to this article.
